# Analysis of Strain Transfer Characteristics of Fiber Bragg Gratings for Asphalt Pavement Health Monitoring

**DOI:** 10.3390/ma18153489

**Published:** 2025-07-25

**Authors:** Zhaojun Hou, Dianguang Cao, Peng Peng, Xunhao Ding, Tao Ma, Jianchuan Cheng

**Affiliations:** 1 School of Transportation, Southeast University, 2 Sipailou, Nanjing 210096, China; 230199132@seu.edu.cn (Z.H.); 230238914@seu.edu.cn (P.P.); matao@seu.edu.cn (T.M.); jccheng@seu.edu.cn (J.C.); 2 CCDI (Suzhou) Exploration & Design Consultant CO., LTD., Suzhou 215000, China; 3 First Harbor Engineering Company LTD., 3 Avenue, Binhai New Area, Tianjin 300457, China; caodianguang@163.com

**Keywords:** fiber Bragg grating, strain transfer, asphalt pavement, strain distribution, structural health monitoring

## Abstract

Fiber Bragg grating (FBG) exhibits strong resistance to electromagnetic interference and excellent linear strain response, making it highly promising for structural health monitoring (SHM) in pavement. This research investigates the strain transfer characteristics of embedded FBG in pavement structure and materials by using the relevant theoretical models. Results indicate adhesive layer thickness and sheath modulus are the primary factors influencing the strain transfer coefficient. A thinner adhesive layer and high modulus of sheath enhance the coefficient. Additionally, the strain distribution of sheath significantly affects the transfer efficiency. When the stress level near the grating region is lower than the both ends, the coefficient increases and even exceeds 1, which typically occurs under multi-axle conditions. As for asphalt mixture, high temperature leads to lower efficiency, while accumulated plastic strain improves it. Although the increased load frequency results a higher strain transfer coefficient, the magnitude of this change is negligible. By employing polynomial fitting to the sheath strain distribution, the boundary condition of theoretical equation could be removed. The theoretical and numerical results of strain transfer coefficient for pavement embedded FBG demonstrate good consistency, indicating the polynomial fitting is adoptable for the theoretical calculation with non-uniform strain distribution. This study utilizes the FEM to clarify the evolution of FBG strain transfer in pavement structures and materials, providing a theoretical basis for the design and implementation of embedded FBG in pavement.

## 1. Introduction

FBG sensors exhibit excellent resistance to electromagnetic interference and provide a highly linear strain response [1,2,3]. With a bare fiber diameter of only 125 μm, the presence of the fiber induces negligible disturbance to the original strain field in the host medium [4]. Currently, FBG is widely employed for critical strain monitoring in key structural components such as phased-array radar systems, aircraft wing skins, and large-scale bridges [5,6,7,8]. In these applications, surface bonding or mounting via a substrate is typically sufficient to ensure reliable strain transfer between the structure and the sensor [9]. However, in pavement structural health monitoring, embedded FBG sensors face numerous issues. High-temperature and high-pressure compaction in construction can threaten sensor survival, while during road service life, coupled vehicular and environmental effects lead to complex internal responses. These factors impose stringent packaging reliability requirements and induce multiple sources of error, affecting strain measurement accuracy [10,11]. To ensure the survival of FBG in pavements, optimization is required in both the fiber’s encapsulation type and the embedding construction process. The use of metal helical mesh or armored FBG sensors significantly reduces the risk of fiber core breakage caused by local shear or compression. Furthermore, during road construction, the method of manual compaction embedding for the pre-treatment of FBG can minimize the damage caused by subsequent roller compaction [12]. For existing roads, slot-cutting and the backfilling embedding method can also avoid the adverse conditions associated with high-temperature and high-pressure construction processes. Additionally, consideration of the embedding survival rate has led to a wide variety of encapsulation forms for FBG in asphalt pavements, each with different impacts regarding its interference in original pavement strain response monitoring [13]. As a result, it is necessary to correct the fiber strain monitoring data in order to obtain the true pavement response.

Analysis of the strain transfer characteristics of fiber Bragg grating can be categorized into three aspects: theoretical solution, numerical simulation, and experimental testing. Among these, theoretical solutions are typically developed based on the assumption of a multilayer elastic structure with the FBG. Ansari et al. [14] introduced a multilayer strain transfer theory to establish a strain transfer model for FBG sensors. By assuming a uniform strain distribution on the outer surface, the attenuation behavior of strain across each encapsulation layer could be derived. Zhou Zhi et al. [15] employed shear lag theory to construct stress equilibrium equations and geometric compatibility differential equations for each layer, incorporating the Goodman assumption to describe the interfacial shear stress transfer in multilayer structures. They obtained an analytical expression for the strain transfer coefficient that accounts for the interface slip between layers. Her S.C. et al. [16] analyzed strain transfer using a three-dimensional model. By neglecting axial stress in the encapsulation layers and the influence of Poisson’s effects in the radial and circumferential directions, the structure was modeled under pure shear, and the layer-by-layer transfer behavior was derived under a uniform strain field. These theoretical studies, based on simplified force transmission and deformation assumptions, have generated various analytical expressions for strain transfer. Notably, the assumption of zero strain at the end of the fiber is valid for surface-bonded circumstances, but it becomes inappropriate for embedded usage, where the fiber is fully bonded to the host medium. In such cases, modified boundary conditions are needed to solve the differential equations governing strain transfer [17].

Additionally, for complex media such as asphalt mixtures and cement-treated aggregate, the grating strain measured is attributed to the material mechanical behavior [18,19]. Wang Qian [20] used a rheological model to characterize the long-term creep period of concrete, considering the stress level, curing age, and mix proportion in evaluating the long-term strain transfer efficiency, and the theoretical analysis was validated by finite element simulation. For cement-treated base, the influence of elastoplastic on strain transfer has been addressed using an equivalent modulus approach [21], where the secant modulus is defined as a function of plastic strain development. For asphalt mixtures, Wang Huaping replaced the elastic modulus in the shear lag model with the Burgers model, enabling the derivation of a strain transfer coefficient that incorporates the viscoelastic properties of the asphalt mixtures.

The numerical method provides a new approach for analyzing strain transfer characteristics of FBG. By constructing a solid fiber–medium coupled geometric model and using the material’s constitutive law, the strain distribution in the fiber core under various loading conditions can be effectively simulated. Zhu Lujia [22] employed ANSYS to investigate the effects of embedded length, coating modulus, and sensor geometry on strain transfer efficiency, and proved the numerical results through specimen tensile tests. Wu Rujun et al. [23] used numerical simulation to obtain the 2D fiber–medium strain transfer, which was compared against the bidirectional coupling analytical model to assess its accuracy. Finite element analysis (FEA) solves the experimental limitations associated with testing embedded fibers in actual pavements and serves as a verification tool for analytical solutions. Furthermore, FEA enables the study of viscoelastic–plastic and damage behaviors of complex materials such as asphalt mixtures and cement-stabilized macadam, offering a feasible and flexible method to evaluate strain transfer efficiency under realistic pavement conditions. The analysis of viscoelastic–plastic and damage behaviors of complex materials such as asphalt mixtures and cement-treated base can be realized by FEA, providing a feasible and flexible method for evaluating strain transfer efficiency under various pavement circumstances.

Existing experimental studies on strain transfer efficiency of FBG have primarily focused on surface-bonded applications. Li Chuang [24] employed a two-point bonding method to attach the grating region on a steel tensile specimen. By comparing the strain measured by the FBG sensor during loading with reference data from a resistive strain gauge, the strain transfer coefficient was obtained. Gao Haichuan [25] utilized a universal testing machine and strain gauges to conduct a comparative analysis of strain sensitivity under different sensor substrate geometries and bonding methods, aiming to guide the selection of substrate configurations for surface-mounted FBG. However, experimental research on embedded FBG strain transfer is relatively limited. In most cases, the strain transfer of embedded FBG is directly applied through the theoretical model. For example, Chen Fengchen [26] used copper tube to encapsulate the fiber for directly monitoring internal strain in asphalt mixtures. Based on an Accelerated Loading Facility, the FBG strain output was used to revise a rutting prediction formula. Dominik Hauswirth et al. [27] embedded distributed optical fibers within pavements through pre-cut grooves and monitored strain variations to evaluate axle load characteristics. Cai Hongliang [28] embedded triaxial FBG sensors within the cement-treated base and the middle asphalt layers during construction period. Through full-scale accelerated pavement loading tests, the dynamic response of the embedded FBG to vehicle traffic was analyzed, demonstrating that FBG maintains good compatibility with pavement materials.

Clarifying the strain transfer characteristics of embedded FBG in pavement is a prerequisite for accurately capturing the true strain response. Considering the temperature sensitivity of asphalt mixtures, non-uniform strain distribution, and the influence of dynamic loading conditions, this study adopts a multilayer shear lag theory to describe the strain transfer of FBG under elastic conditions. In addition, a combination of polynomial fitting and numerical simulation is employed to analyze the influence of multiple factors on the strain transfer efficiency of embedded FBG. The theoretical solutions are validated within a pavement-scale simulation model, providing theoretical support and practical guidance for the design and application of embedded FBG structural health monitoring systems in asphalt pavement.

The structure of this article is organized as follows: Section 2 outlines the objectives of this study, which aims to clarify the evolution of strain transfer efficiency in FBG used for pavement monitoring. Section 3 introduces the theoretical method for solving the strain transfer coefficient of FBG, the viscoelastic–plastic behavior of asphalt mixtures that influence strain transfer efficiency, and the cylindrical specimen and pavement structure models used for numerical simulations. These models are used to analyze the various factors affecting the strain transfer efficiency in FBG. Section 4 examines the influence of fiber structure factors, external factors such as load and temperature, and the effects of material and pavement scales on the variation of strain transfer efficiency. Finally, Section 5 presents the main conclusions, which can be used to guide data correction for FBG-based strain monitoring of asphalt pavements.

## 2. Objective

In this research, FBG with braided metal armor is selected as the representative structure. Through a combination of numerical simulation and theoretical analysis, the evolution characteristics of the strain transfer in asphalt mixture and structure are investigated, aiming to clarify the influence mechanisms of fiber structural parameters, strain distribution patterns, and load–temperature coupling effects on strain transfer efficiency under pavement conditions. This research supports the application of FBG in strain monitoring of cement-treated base and asphalt base layers, as well as in rutting monitoring of the middle and lower asphalt layers.

## 3. Methodology

### 3.1. Strain Transfer Theory of Fiber Bragg Grating

When a broadband optical spectrum propagates through the fiber core, the periodic grating structure in the core of the optical fiber induces Bragg reflection at a specific wavelength. As the grating undergoes deformation due to external environmental changes, the center wavelength of the reflected light shifts linearly, enabling the monitoring of strain. By demodulating the reflected wavelength signal using an optical interrogator, the strain in the grating can be quantitatively obtained, thus realizing the strain sensing function of the FBG. The relationship between grating strain ε and the Bragg reflected wavelength λ is given by Equation (1):

(1) $$\varepsilon =\frac{\Delta \lambda }{(1-P_{e})\lambda }$$

where $P_{e}$ denotes the photoelastic coefficient related to the fiber core material. For the germanium-doped silica, $P_{e}$ is approximately 0.22 at normal temperature.

In this study, a braided metal armor FBG is employed, with a grating length of 10 mm. The sensor structure consists of a tightly buffered fiber composed of a fiber core, coating layer, and adhesive layer; spiral steel reinforced wire mesh; and PVC outer sheath. This design ensures mechanical durability and environmental resistance under the complex stress and load conditions in asphalt pavement. Table 1 summarizes the geometrical dimensions and basic material properties of the braided metal armor FBG.

**Figure 1:**
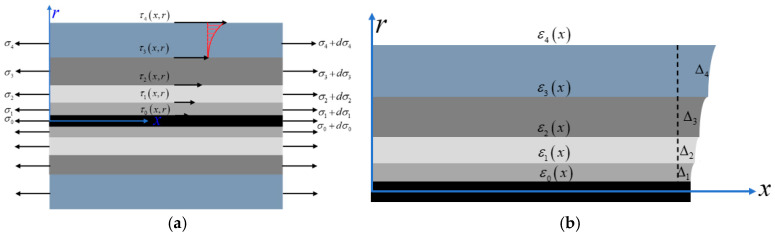
Shear lag theory for multilayer encapsulated FBG. (**a**) Stress distribution of FBG. (**b**) Deformation pattern of FBG.

The presence of encapsulation layers leads to a discrepancy between the strain of host medium and grating region. Therefore, it is necessary to analyze the strain transfer behavior of the multilayer encapsulated structure to correct the measured strain. For semi-rigid base such as cement-treated aggregates, the materials remain at an elastic stage for the majority of service life. In this way, shear lag theory is employed to analyze the strain transfer behavior of the armored FBG under elastic conditions. Figure 1 illustrates the internal stress and strain distribution on the cross-section of the grating region subjected to axial deformation.

When the host medium undergoes an axial deformation, each encapsulation layer of the fiber is subjected to shear stress and axial stress. The shear stress within the encapsulation layers varies along the radial direction, leading to a gradual increase in axial strain toward the fiber core as the radial position *r* decreases. This strain gradient results in a mismatch between the strain level of the monitored medium and that of the grating region. By performing force equilibrium analysis on a differential grating segment *dx*, the following expression is derived as Equation (2a), and by recursive formulation, Equation (2b) is obtained:

(2a) $$\tau_{i}\left(x,r\right)=\frac{r_{i-1}}{r}\tau_{i-1}-\frac{r^{2}-r_{i-1}^{2}}{2r}\frac{d\sigma_{i}}{dx}$$

(2b) $$\tau_{i}\left(x,r\right)=-\frac{1}{2r}\sum_{j=0}^{i-1}\left(r_{j}^{2}-r_{j-1}^{2}\right)\frac{d\sigma_{j}}{dx}-\frac{1}{2r}\left(r^{2}-r_{i-1}^{2}\right)\frac{d\sigma_{i}}{dx}$$

In the above equations, *i* = 0~4 denotes the index of each structural layer, and *r*_−1_ is defined as the radial position of the fiber core. $\tau_{i}$ represents the shear stress in the *i*-th layer, and $d\sigma_{i}$ denotes the increment axial stress within a differential segment of the *i*-th layer.

Based on the definition of shear stress $\tau_{i}\left(x,r\right)$, the shear deformation along the radial direction in each encapsulation layer can be expressed as Equation (3):

(3) $$\Delta_{i}=\int_{r_{i-1}}^{r_{i}}\frac{\tau_{i}\left(x,r\right)}{G_{i}}dr$$

where $G_{i}=\frac{E_{i}}{2\left(1+\gamma_{i}\right)}$ denotes the shear modulus of the *i*-th encapsulation layer.

Considering the radial dimension of the fiber structure (2.7 mm) is much smaller than its length, and the grating is primarily subjected to axial loading, the radial and circumferential stresses can be neglected. Based on the constitutive equations of elasticity, the following relationship can be obtained:

(4) $$\frac{d\sigma_{i}}{dx}=E_{i}\frac{d\varepsilon_{i}}{dx}$$

The compatible deformation between encapsulation layers allows for the assumption that the axial strain gradient exhibits a consistent trend across all layers, $\frac{d^{2}\varepsilon_{i+1}}{dx^{2}}\approx \frac{d^{2}\varepsilon_{i}}{dx^{2}}$. Meanwhile, when the host medium undergoes deformation, the displacement relationship between the outer sheath and the fiber core follows Equation (5a). And the displacement difference between the two values is generated by the shear deformation differences across the encapsulation layers, as shown in the geometric relationship in Figure 1b. By differentiating the displacement values, the differential relationship between the surface strain of the host medium and fiber core can be derived, as shown in Equation (5b).

(5a) $$U_{4}\left(x\right)-U_{0}\left(x\right)=\sum_{i}\Delta_{i}\left(x\right)$$

(5b) $$\varepsilon_{4}\left(x\right)-\varepsilon_{0}\left(x\right)=\sum_{i}\int_{r_{i-1}}^{r_{i}}\frac{\dot{\tau }\left(x,r\right)}{G_{i}}dr$$

By substituting Equations (2b) and (4) into Equation (5b), and applying the simplified assumption of consistent axial strain gradient evolution, the following expression is obtained:

(6) $$\varepsilon_{4}\left(x\right)-\varepsilon_{0}\left(x\right)=-k^{2}{\ddot{\varepsilon }}_{0}\left(x\right)$$

where $k^{2}=\sum_{i=1}^{4}\frac{1}{G_{i}}\left[\sum_{j=0}^{i-1}\left(\frac{r_{j}^{2}-r_{j-1}^{2}}{2}E_{j}\ln\left(\frac{r_{i}}{r_{i-1}}\right)\right)+\left(\frac{r_{i}^{2}-r_{i-1}^{2}}{4}-\frac{r_{i-1}^{2}}{2}\ln\left(\frac{r_{i}}{r_{i-1}}\right)\right)E_{i}\right]$ is a coefficient related to the geometry and material properties of FBG.

To solve differential Equation (6), appropriate boundary conditions must be introduced. For surface-bonded fibers, the strain is zero at the end of bonded segment. However, the outer surface of the fiber is in intimate contact with the host medium for embedded fibers, and the concept of effective bonding length should be applied. The external forces beyond the effective bonding length has less influence for the strain transfer within the grating region. The effective bonding length of the embedded FBG is denoted as ±nL, and the weakened boundary condition is defined as $\varepsilon_{0}\left(\pm nL\right)=0$. Due to vehicle movement, the strain level within the road changes over time. Since the FBG is an elastic structure that exhibits time independence, the time index introduced in the above theoretical derivation is used to represent the strain level at given moment *t*. Additionally, the explicit relationship between the axial strain in the grating region and the surface strain of the host medium can be derived, along with the expression for the average strain transfer coefficient α within the grating length.

(7a) $$\varepsilon_{0}\left(x,t\right)=\varepsilon_{4}\left(t\right)\left(1-\frac{\cosh\left(\frac{x}{k}\right)}{\cosh\left(\frac{nL}{k}\right)}\right)$$

(7b) $$\alpha =\frac{\int_{-0.5L}^{0.5L}\varepsilon_{0}\left(x,t\right)dx}{L\varepsilon_{4}\left(t\right)}=1-\frac{k\sinh\left(\frac{0.5L}{k}\right)}{0.5L\cosh\left(\frac{nL}{k}\right)}$$

**Figure 2:**
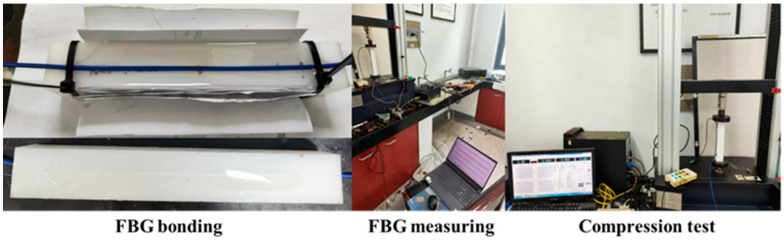
FBG strain transfer testing process.

To verify the accuracy of the theoretical formulas, this study used a low-density rectangular polyethylene (LDPE) prism with dimensions 20 × 3 × 3 cm as the monitoring material. Armored FBG and bare FBG, as shown in Table 1, were arranged on two opposite sides of the specimen. These FBGs were fixed to the surface using epoxy resin, with a bonding length of 14 cm. Compression tests were conducted using a 5000 N capacity tensile–compressive testing machine to obtain the strain transfer efficiency of both types of FBGs. Figure 2 shows the process flow of the FBG compression test.

### 3.2. Strain Transfer Behavior of Fiber Bragg Grating with Viscoelastic Response

For viscoelastic materials such as asphalt mixtures, the surface strain $\varepsilon_{4}\left(x,t\right)$ exhibits a history-dependent response to applied stress. According to the Boltzmann linear superposition principle [29], the relationship can be expressed in the form of Equation (8):

(8) $$\varepsilon_{4}\left(x,t\right)=\int_{0}^{t}E\left(\frac{t-\tau }{a_{T}^{ve}}\right)\frac{d\sigma_{4}\left(x,t\right)}{d\tau }$$

where E is the relaxation modulus; *τ* is used as the integration variable and represents relaxation time. $\sigma_{4}\left(x,t\right)$ represents the axial stress in the region near the surface of host medium, and $a_{T}^{ve}$ denotes the temperature factor determined using the Arrhenius Equation (9).

(9) $$a_{T}^{ve}=e^{\theta_{ve}\left(\frac{1}{T}-\frac{1}{T_{ref}}\right)}$$

where $\theta_{ve}$ is the temperature shift factor, and $T_{ref}$ represents the reference temperature.

To investigate the strain transfer characteristics of FBG embedded in asphalt mixtures under dynamic loading, a numerical model of cylindrical asphalt specimen with a diameter and height of 150 mm was established. The multilayer encapsulated FBG was embedded at the center of the specimen, with the grating region located at the mid-height position. A semi-sine load waveform with frequency ranging from 5 to 20 Hz, coupled with a temperature range of 0~35 °C, was applied to analyze the evolution trend of the strain transfer coefficient under viscoelastic conditions. To ensure sufficient data resolution while balancing model size, a non-uniform meshing strategy was adopted. The hexahedral mesh was refined near the fiber and gradually enlarged outward from the specimen center. The mesh size was set to 0.5 mm to ensure adequate data resolution at the grating region along the vertical direction. Moreover, the viscoelastic behavior was characterized using a Prony series, expressed in Equation (10), with the material parameters fitted from AC-20 dynamic modulus test data [30]. Table 2 presents the viscoelastic parameters and the corresponding WLF temperature shift coefficient used in the model.

(10) $$E\left(t\right)=E_{\infty }+\sum_{i}E_{i}e^{-\frac{t}{\rho_{i}}}$$

where $E_{\infty }$ is the equilibrium modulus, $E_{i}$ is the relaxation modulus components, and $\rho_{i}$ denotes the corresponding relaxation times.

Under axial cyclic compression loading applied to the cylindrical specimen, the outer encapsulation layer exhibits uniform axial strain distribution $\varepsilon_{4}\left(x,t\right)$ at any time. Additionally, since all encapsulation materials are linear elastic, the strain transfer coefficient under viscoelastic response can be directly calculated using Equation (7b). However, in pavement structures, the internal strain level $\varepsilon \left(x,t\right)$ of the host medium varies with the proximity of vehicular axle loads, increasing as the load approaches and decreasing as it moves away. Moreover, the strain at different positions are non-uniformly distributed. Based on shear lag theory, for multilayer encapsulated FBG, the outer sheath and the fiber core are related by the second-order constant coefficient linear non-homogeneous differential equation, as shown in Equation (6). Furthermore, under vehicle movement, the horizontal strain distribution in the transverse and longitudinal directions within the road structure can be fitted using a power function. When the strain distribution in the outermost layer of the FBG is fitted with a polynomial function, the strain distribution in the fiber core follows the same form and order. The distribution functions for both can be expressed as Equation (11). By substituting Equation (11) into the differential form of Equation (6), the strain transfer can be solved without introducing boundary conditions, making it consistent with practical embedding of FBG in pavement. Equation (12) provides the expression for the polynomial coefficients of the grating strain, which can be used to compute the strain transfer coefficient under non-uniform strain distribution.

(11) $$\left\{\begin{array}{c} \varepsilon_{4}\left(x,t\right)=\sum_{i=0}^{n}a_{i}x^{i}\\ \varepsilon_{0}\left(x,t\right)=\sum_{i=0}^{n}b_{i}x^{i} \end{array}\right.$$

where $a_{i},b_{i}$ represents the polynomial fitting coefficients. For the strain distribution within the cement-treated base layer, a fifth-order polynomial function can be used for fitting. However, for the asphalt-stabilized layer, due to the viscoelastic response, the strain distribution is more complex. Therefore, a ninth-order polynomial function is used for ATB.

By substituting Equation (11) into differential Equation (6), the fitting coefficients for the fiber core can be obtained, as shown in Equation (12). The coefficients $b_{j}$ and $a_{j}$ follow a recursive relationship.

(12) $$\left\{\begin{array}{l} b_{j}-k^{2}b_{j+2}\left(j+2\right)\left(j+1\right)=a_{j}\;\;j=0\sim n-2\\ b_{j}=a_{j}\;\;\;\;\;\;\;\;\;\;\;\;\;\;\;\;\;\;\;j=n,n-1\; \end{array}\right.$$

**Figure 3:**
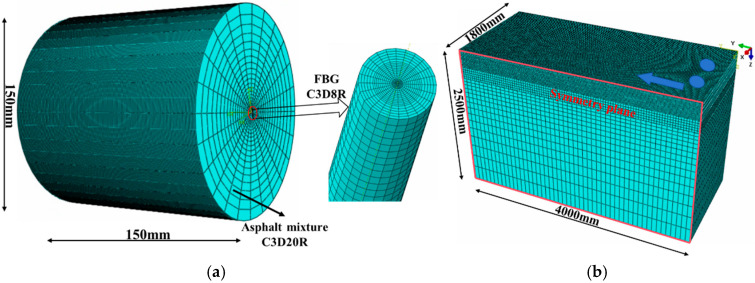
Numerical models for viscoelastic response of fiber Bragg grating. (**a**) Embedded FBG cylindrical specimen model. (**b**) Pavement structure model.

Furthermore, to investigate the strain transfer characteristics of FBG under pavement structures, a representative asphalt-stabilized base pavement was established, detailed in Table 3. Under a standard dual-wheel uniaxial dynamic load, the time history strain response at the bottom of the base was extracted and subsequently imposed as a displacement boundary condition on the outer surface of the embedded fiber. This approach enables the analysis of strain transfer efficiency under varying strain distributions. Figure 3 illustrates the cylindrical specimen and pavement structure used in viscoelastic strain transfer analysis.

### 3.3. Strain Transfer of Fiber Bragg Grating Under Plastic Deformation

The permanent deformation behavior of asphalt mixtures causes irreversible changes in the strain levels of embedded FBG, and the unevenly distributed plastic strain along the grating region also affects strain transfer efficiency. To clarify the impact of permanent deformation in asphalt mixtures on the strain level variation and transfer efficiency of FBG, this study introduces plastic behavior in the numerical calculation model shown in Figure 3a, which is achieved by applying high-temperature, high-pressure static loads to generate different levels of permanent deformation. To address this, the modified Mohr–Coulomb yield criterion proposed by Sun et al. [31] is adopted, and the influence of shear stress is represented by the second invariant of the deviatoric stress tensor, while the first invariant of the stress tensor is used to characterize the confining pressure.

(13) $$f=R_{mc}\sqrt{J_{2}}-\frac{1}{3}I_{1}\tan\phi -c$$

where $R_{mc}$ is the Mohr–Coulomb coefficient, determined by the Lode angle in 3D stress space and the internal friction angle ϕ of the asphalt mixture. c represents the cohesion coefficient of asphalt mixture, which characterizes material’s resistance to plastic deformation and is governed by the viscoplastic equivalent strain $\varepsilon_{ij}^{vp}$.

The plastic flow direction of asphalt mixture is defined as the gradient of a plastic potential function and can be expressed as Equation (14).

(14) $${\dot{\varepsilon }}_{ij}^{vp}=a_{T}^{vp}\Gamma^{vp}f^{N}\frac{\partial g}{\partial \sigma_{ij}}$$

where $a_{T}^{vp}$ is the plastic temperature shift factor and shares the same functional form as $a_{T}^{ve}$. $\Gamma^{vp},N$, determined through repeated creep tests, are the viscoplastic material parameters. $g=\sqrt{J_{2}}-\alpha I_{1}$ represents the plastic potential function, used to determine the direction of the plastic strain tensor.

When the internal stress reaches the plastic yield surface, permanent plastic deformation occurs, as defined by Equation (14). Meanwhile, the accumulation of plastic strain leads to an increase in the cohesion coefficient c [32], resulting in an evolution of the yield surface. This reflects the hardening behavior of the asphalt mixture, which is characterized by the change in cohesion expressed by Equation (15).

(15) $$c=a_{T}^{c}\left(c_{0}+c_{1}\left(1-e^{-c_{2}\varepsilon_{e}^{vp}}\right)\right)$$

where $a_{T}^{c}$ is the temperature shift factor for cohesion. $c_{0},c_{1},c_{2}$ are the cohesion hardening parameters, used to represent the initial cohesion, ultimate cohesion, and the hardening rate of asphalt mixtures. $\varepsilon_{e}^{vp}=\sqrt{\frac{2}{3}\varepsilon_{ij}^{vp}\varepsilon_{ji}^{vp}}$ denotes the equivalent plastic strain, which characterizes the level of plastic deformation at a given point.

The three equations above fully describe the plastic behavior of asphalt mixtures, and the UMAT subroutine is used for numerical calculations. Table 4 summarizes the material parameters used in the plasticity model for the asphalt mixtures. This study controls the generation of plastic strain levels within the range of 0 to 3000 *με* for cylindrical specimens, representing the entire pavement rutting evolution process. It also analyzes the variation in strain transfer efficiency of FBG under different plastic strain levels and distribution forms, aiming to guide the data correction for FBG-based monitoring of rutting in pavement.

## 4. Results and Discussion

### 4.1. Sensitivity Analysis of Strain Transfer Parameters for Fiber Bragg Grating

**Figure 4:**
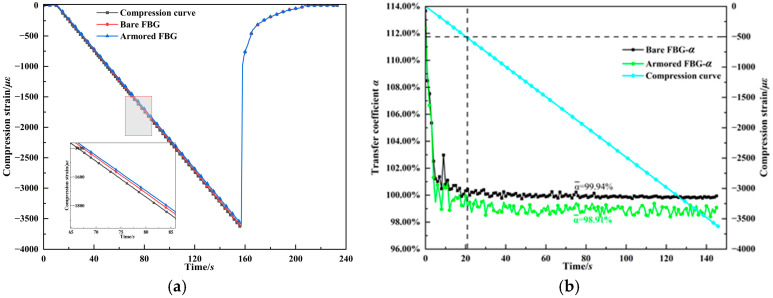
FBG compression testing experiment. (**a**) Compression strain curve. (**b**) Strain transfer coefficient α. The black dashed lines are used to indicate the α calculation range.

Figure 4 shows the results of the surface-mounted FBG compression testing experiment. LDPE is a super-elastic material with a nonlinear stress–strain relationship. To ensure that the strain increases linearly during compression loading, facilitating the calculation of the strain transfer coefficient, a displacement-controlled loading mode was used during the test, with a loading rate of 0.3 mm/min and a compression loading duration of two and a half minutes. Figure 4a presents the strain response process of the two types of FBGs (0–233 s) and the displacement loading segment recorded by the tensile–compressive testing machine (11–155 s). Within the loading segment, the FBGs show a good linear increase, and the inset magnified view reveals that the strain monitored by the bare FBG is closer to the compression curve, showing a higher strain transfer efficiency. Figure 4b shows the strain transfer coefficients of the two types of FBGs under different compressive strains. The results indicate that at the initial stage of compression loading, the strain transfer coefficient of the FBG fluctuates significantly. On one hand, this may be due to the instability of the contact surface between the monitoring medium and the pressure plate at the start of loading. On the other hand, the frequency limitations of the demodulator and the testing machine data recording also influence the measurement. The calculation of the coefficient uses measurement data recorded at the closest integer time points, and, particularly at low strain levels, errors due to data deviations can lead to distortion of the results. It is noteworthy that when the strain exceeds 500 με, the fluctuations in the transfer coefficient due to time alignment issues are significantly reduced. Calculating the average strain transfer coefficient using all data points can further minimize the impact of such random errors. The average strain transfer coefficient for the bare FBG is 99.94%, with almost no strain loss. The average strain transfer coefficient for the armored FBG is 98.91%, which is very close to the transfer coefficient of 99.08% calculated from the theoretical formula (Equation (7b)) for a ±7L bonding length. These FBG strain calibration tests indicate that the theoretical calculation formula used in this study is correct.

**Figure 5:**
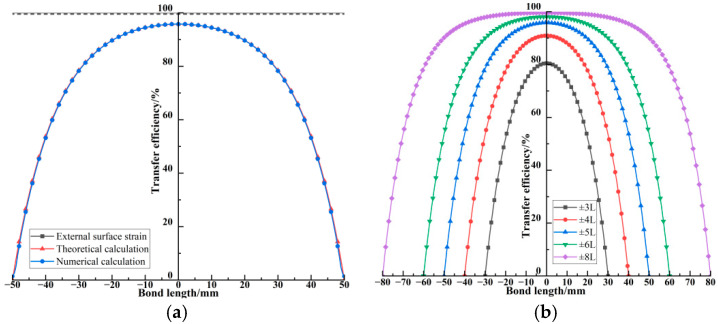
Strain transfer efficiency of the fiber core. (**a**) Theoretical and numerical calculation. (**b**) Effect of bonding length on strain transfer.

Figure 5a illustrates the strain distribution in the fiber core segment calculated by both theoretical formulation and numerical simulation when the outer sheath is subjected to a uniform strain of 100 *με*. Theoretical calculation results are derived from Equation (7a), which is based on the shear lag theory and represents an analytical solution. Numerical calculation results are obtained from finite element analysis, where uniform strain is applied to the outer surface of the multilayer encapsulated FBG with a length of 100 mm, providing a numerical solution. The illustrated results show that the strain distribution in the fiber core segment obtained from both methods is essentially consistent, with the main deviation occurring at the positions at both ends, thereby validating the accuracy of the theoretical formula. The strain transfer efficiency in the fiber core exhibits a bell-shaped distribution, with the highest value at the center of the grating region, reaching 95.80%, and slightly lower values at the grating ends, reaching 95.48%. At the fiber extremities ±5*L*, the strain transfer efficiency drops to zero due to the boundary conditions. The theoretical and numerical results are in great agreement. Figure 5b compares the effects of different bonding lengths. Due to the free boundary condition at the bonding section ends, the strain at the fiber core extremities is zero, and the strain transfer efficiency improves with the bonding length increasing. This improvement is particularly significant for short bonding lengths, with a bonding length of ±3*L*, the center strain transfer efficiency is 80.49%, while when the bonding length is extended by 20 mm, the center efficiency increases to 90.92%. Moreover, When the bonding length exceeds 100 mm, the maximum efficiency surpasses 95%, indicating that bonding length is a critical factor for strain transfer efficiency. The increase in bonding length causes the bell-shaped distribution to flatten, with the strain attenuation on both sides of the grating region slowing down. Therefore, it is essential to determine the effective bonding range for embedded FBG.

**Figure 6:**
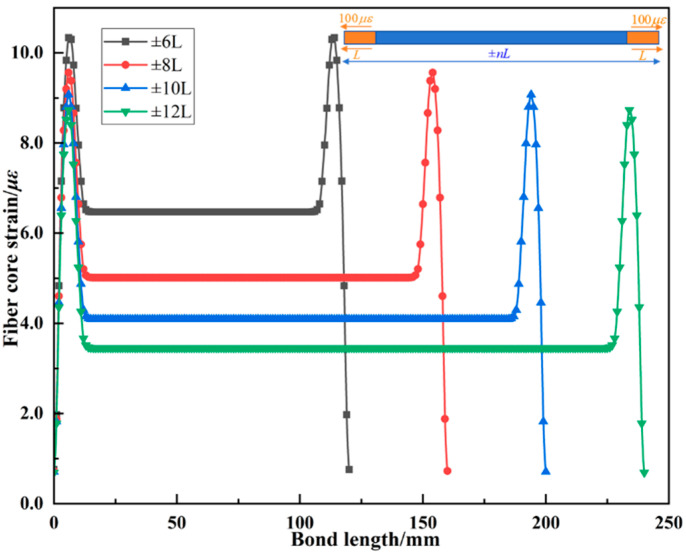
Analysis of effective bonding length for embedded optical fiber.

To further investigate the effect of embedded fiber length on the strain measured within the grating region, Figure 6 presents the strain distribution of the fiber core with a uniform strain of 100 *με*, applied over the outer sheath within a length of *L*, under various total fiber lengths (±*nL*). The results indicate strain variation in the fiber core is most pronounced within the loaded segment *L*, and the influence of boundary end strain diminishes as the fiber length increases. Additionally, under a length of ±6*L*, the contribution of boundary end strain to the grating region reaches 6.47%. When the fiber length increases to ±12*L*, this contribution decreases to 3.45%. Since the strain levels inside a pavement structure are typically less than 70 *με*, the fiber segment length at which the strain contribution from distant ends falls below 5% can be regarded as the effective bonding length in material-scale tests and treated as the dominant strain-contributing segment in pavement. According to the results in Figure 6, the effective bonding length for embedded optical fibers can be taken as approximately ±7*L*.

**Figure 7:**
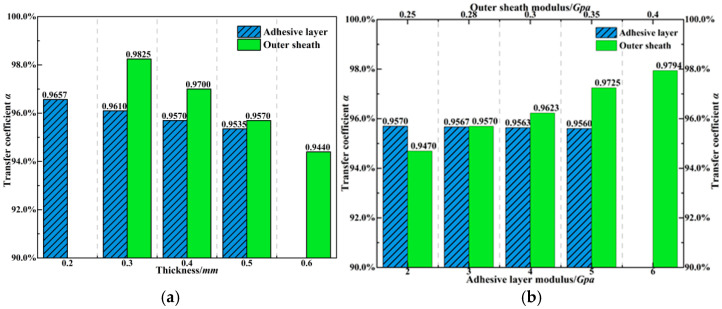
Influence of encapsulation layer on FBG strain transfer. (**a**) Analysis of encapsulation layer thickness. (**b**) Analysis of encapsulation layer modulus.

$k^{2}$ represents the effect of material thickness and properties of each encapsulation layer. Considering the manufacturing constraints of FBG, the fiber core, coating layer, and braided armor layer have well-defined material properties and minimal thickness variation, and the core and coating layer are responsible for monitoring, while the braided armor provides mechanical protection. Therefore, the strain sensitivity of the grating is primarily controlled by the thickness and material selection of the adhesive layer and outer sheath. Figure 7 presents the influence of these four key factors on the strain transfer efficiency of the fiber core segment under a total fiber length of ±5*L*. Both the adhesive layer and outer sheath thicknesses reduce the strain transfer efficiency, and a linear relationship is observed between the strain transfer rate and the thickness of these layers. Notably, the strain sensitivity to outer sheath thickness is 3.2 times higher than the adhesive layer; an increase of 0.1 mm in the outer sheath results in a 1.2% decrease in strain transfer efficiency. In addition, the modulus of the adhesive and outer sheath has different effects on strain transfer. Increasing the adhesive layer modulus slightly reduces the transfer rate, while an increase in the outer sheath modulus significantly improves strain transfer efficiency. Therefore, thinner and high-modulus outer sheaths are recommended for embedded FBG.

**Figure 8:**
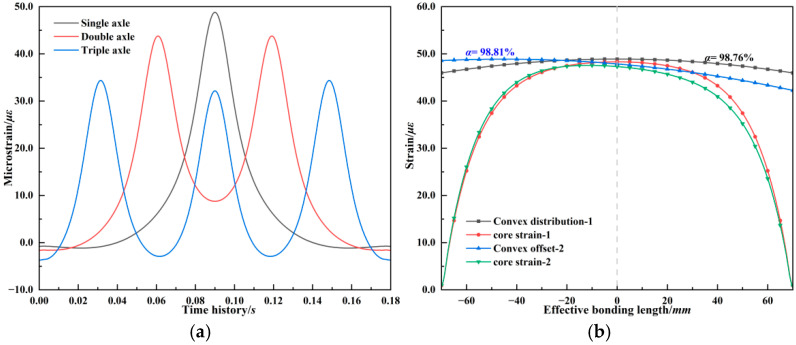

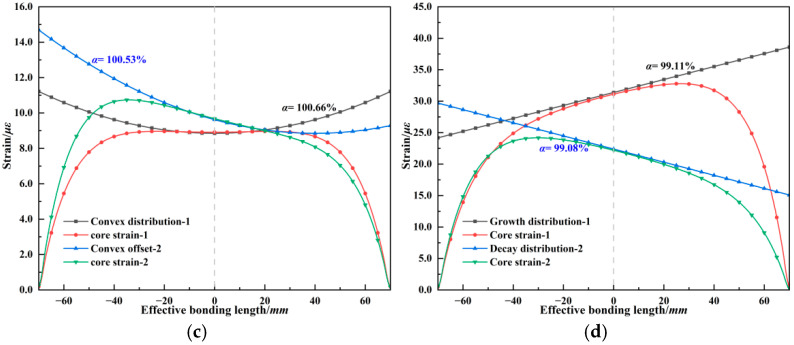
Analysis of fiber strain transfer efficiency under non-uniform strain distribution. (**a**) Strain evolution under moving axle loads. (**b**) Convex distribution. (**c**) Concave distribution. (**d**) Monotonic distribution.

The strain transfer efficiency discussed above was obtained under uniformly distributed surface strain. However, the internal strain distribution within pavement structures changes with the position of the load. The influence of non-uniform strain distributions on the accuracy of strain monitoring must be clarified. Figure 8 shows the strain responses at a point located at the bottom of asphalt base with elastic pavement structure under single, dual, and tridem axle loading, with speed of 60 km/h. The internal strain increases and then decreases as the load passes. The strain response exhibits multiple peaks and valleys under dual and tridem axle loading. And the overlapping of tensile and compressive stresses with multi-axle loads reduces the strain peak due to axle interaction. These loading conditions produce three typical strain distribution patterns within the fiber’s effective bonding length ±7*L*: (1) a convex parabolic curve where strain increases then decreases, (2) a concave parabolic curve where strain decreases then increases, and (3) a monotonic curve with continuous increase or decrease. The boxed regions in Figure 8 highlight these three distribution types. To evaluate strain transfer efficiency under non-uniform strain fields, these three typical strain profiles were applied to the outer surface of the fiber as displacement boundary conditions. Figure 8b corresponds to the convex parabolic case, which occurs when the vehicle load is near the center of the fiber segment. In this case, the monitored strain is highest, and the grating strain is slightly lower than the surface strain. As the convex parabola shifts, deviations appear in the core strain distribution. Figure 8c shows the strain distribution and transfer coefficient for the outer sheath and fiber core under multi-axial loading. In this case, the strain distribution within the effective bonding length of the outer sheath is concave, and the strain level in the corresponding monitoring medium for the grating region is lower than that at the ends. The higher strain levels at both ends contribute more to the strain in the grating region compared to a uniform strain distribution, resulting in a slightly higher strain level in the grating region than the strain level in the corresponding 10 mm host medium. This leads to a strain transfer coefficient greater than 1. Figure 8d shows the monotonic curve, which occurs as the vehicle approaches or leaves the fiber region. The fiber core strain distribution shows a waveform shift within the effective bonding segment. Among the three, the monotonic distribution yields a strain transfer efficiency close to the theoretical value under uniform strain. The convex profile results in a slightly reduced efficiency, while the concave profile under multi-axle loads may cause the transfer efficiency to exceed 1.

### 4.2. Strain Transfer Analysis of Fiber Bragg Grating Embedded in Asphalt Mixture

**Figure 9:**
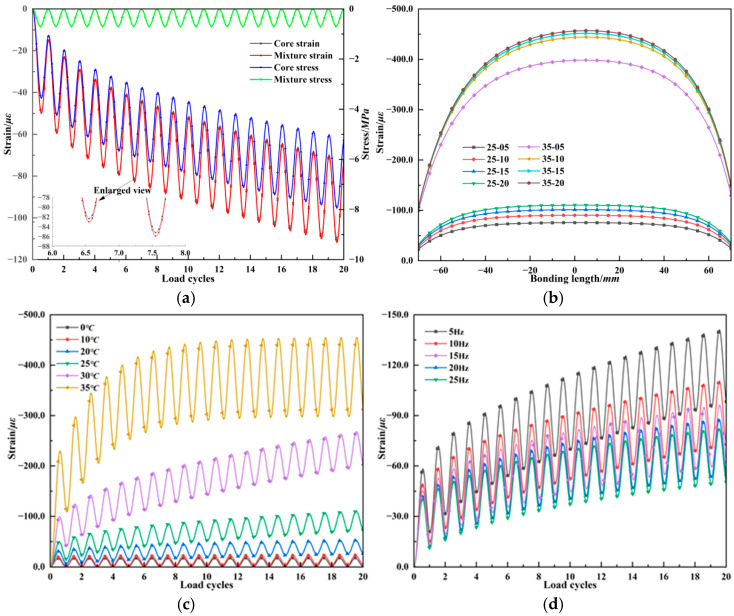
FBG strain response under dynamic loading. (**a**) Strain response under 10 Hz and 25 °C. (**b**) Strain distribution. (**c**) Temperature effect on FBG. (**d**) Frequency effect on FBG.

When FBG is used for strain monitoring in asphalt mixture, the viscoelastic behavior causes both the magnitude and distribution of strain in the host medium, which in turn affects the strain level of grating region. To assess the performance of FBG in the strain monitoring of asphalt-stabilized base, Figure 9 presents the fiber strain response characteristics under dynamic loading in an asphalt mixture cylindrical specimen. Under a conventional 10 Hz half-sine load and at 25 °C, the stress–strain responses of host medium and the fiber core show good agreement, indicating the embedded FBG can accurately reflect the actual strain state of the asphalt mixture. Moreover, while the asphalt mixture is subjected to a constant 0.7 MPa half-sine load, the fiber core exhibits increasing stress levels with each load cycle due to the purely elastic nature of the fiber that lacks stress relaxation. Figure 9b shows the strain distribution within the effective bonding length of the fiber at different loading cycles. Strain levels increase with the number of load cycles, and strain near the ends of the specimen is lower than the center, forming a convex curve. At high temperatures, both the strain magnitude and distribution change significantly with the number of loading cycles. After about ten cycles, the strain distribution stabilizes and the convex curve becomes more pronounced, leading to a reduction in the strain transfer efficiency of the grating region. As shown in Figure 9c, the strain growth in the fiber core converges and reaches a stable state more quickly under higher temperatures. At 35 °C, the maximum strain in the fiber core reaches 456.18 *με*, which is well within the ±5000 *με* measurement range of the FBG. These results demonstrate that the viscoelastic behavior of asphalt mixtures leads to increasing stress and strain levels in the fiber core under dynamic loading, which differs from the response of elastic materials such as cement-treated bases. Moreover, Figure 9d presents the fiber core strain response under different loading frequencies at 25 °C. The strain in the fiber core also increases with the load cycles increases, and a lower frequency load leads to a more pronounced growth trend.

**Figure 10:**
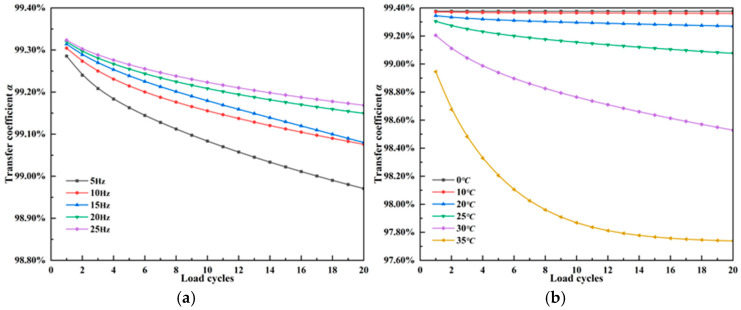
Strain transfer coefficient of fiber Bragg grating under viscoelastic response. (**a**) Effect of loading frequency. (**b**) Effect of temperature.

Figure 10 presents the strain transfer coefficient of the FBG corresponding to the peak strain moments during cyclic loading. At a normal pavement temperature of 25 °C, higher dynamic loading frequencies result in a slightly increased strain transfer coefficient. However, the variation in transfer efficiency due to frequency is not significant. The frequency change from 5 Hz to 25 Hz only increases the strain transfer coefficient by 0.25%, as the mechanical behavior of asphalt mixtures at this temperature is primarily elastic. Given that traffic loading frequencies on asphalt pavements typically range from 5 to 15 Hz, the influence of loading frequency on the accuracy of fiber strain monitoring can be considered negligible under normal temperature. At a fixed loading frequency of 10 Hz, temperatures below 25 °C do not significantly affect the strain transfer coefficient, which can reach over 99.3%. For normal vehicle operation on pavements at standard temperature, the FBG monitoring strain can be considered to reflect the truth dynamic strain of the pavement, while an increase in temperature leads to a marked reduction in transfer efficiency. Under high-temperature conditions (e.g., 35 °C), the strain transfer coefficient experiences a rapid and substantial decline over a short period. Therefore, for summer pavement monitoring at high temperatures, it is necessary to apply a correction to the FBG strain data using a smaller strain transfer coefficient. Particularly, the coupling of high temperature and low frequency loads further exacerbates the reduction in strain transfer coefficient. According to the time–temperature equivalence principle in Equation (9), taking 25 °C as the reference temperature for typical asphalt-stabilized base, the viscoelastic response of the asphalt mixture can be neglected when the dynamic loading frequency exceeds 5 Hz. However, in summer, where the temperature exceeds 35 °C, the influence of viscoelastic effects on strain transfer coefficient must be considered even under standard vehicle operating speeds.

**Figure 11:**
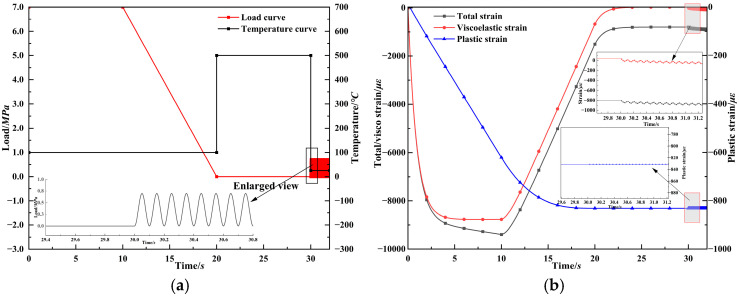
Viscoplastic strain monitoring analysis of asphalt mixture. (**a**) Load–temperature control curve. (**b**) Strain response of asphalt mixture.

The viscoelastic analysis of fiber strain response presented above does not account for the effects of plastic deformation in asphalt mixtures. When applied to asphalt surface layers, the plastic strain distribution of the layer must be considered. Figure 11 illustrates the load–temperature curve used to control permanent deformation levels in the asphalt specimen and the corresponding strain responses. During the initial stage (0–10 s), high-temperature and high-pressure conditions are applied to induce permanent deformation. Subsequently, a high-pressure unloading stage (10–20 s) is utilized to facilitate the rapid dissipation of internal stress and strain. Considering the viscoelastic behavior of asphalt mixtures, a temperature ramping stage (20–30 s) is also adopted to accelerate the dissipation of residual stress. Finally, the dynamic response frequency with a 10 Hz cyclic load is applied during the last stage (30–32 s), under three temperature conditions—0 °C, 25 °C, and 45 °C—representing low, moderate, and high seasonal temperatures, respectively. Figure 11b presents the strain response of the asphalt mixture corresponding to the grating region. The viscoelastic strain increases rapidly under high-temperature static loading and gradually stabilizes. It then decays during the unloading and temperature increase phases, approaching zero. Meanwhile, the plastic strain accumulates linearly during the high-pressure phase and continues to grow with a slower rate during unloading, eventually stabilizing. It is worth noting that the plastic strain remains constant during the subsequent dynamic loading phase. In addition, the total strain response of the specimen reflects the viscoelastic strain and with an offset corresponding to the accumulated plastic strain. Meanwhile, the strain measured in the grating region results from both the plasticity and viscoelasticity of the host medium. During the dynamic loading phase (30–32 s), the strain in the grating region appears as a shifted version of the viscoelastic strain curve due to the superimposed plastic strain. Since the plastic strain remains steady, the viscoelastic strain transfer coefficient $\alpha_{ve}$ can be defined as the ratio of the mean change in strain in the grating region to the peak viscoelastic strain of the specimen $\Delta {\bar{\varepsilon }}_{e}/{\bar{\varepsilon }}_{ve}$. Similarly, the plastic strain transfer coefficient $\alpha_{p}$ is defined as the ratio of the mean grating strain to the specimen’s plastic strain ${\bar{\varepsilon }}_{e}/{\bar{\varepsilon }}_{p}$.

**Figure 12:**
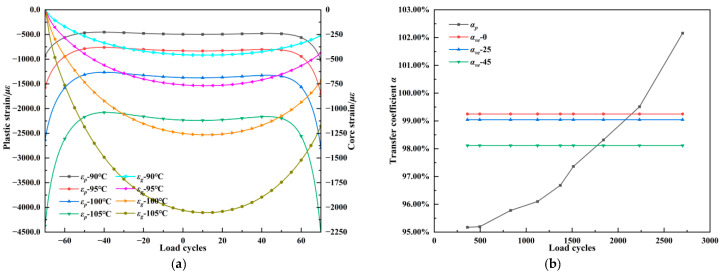
Strain transfer of the embedded FBG under plastic deformation. (**a**) Strain distribution on the outer sheath. (**b**) Strain transfer coefficient under different plastic deformation.

To clarify the influence of plastic strain magnitude and distribution on the strain transfer coefficients for viscoelastic and permanent deformation monitoring, Figure 12a presents the fiber core strain and the plastic strain distribution of the asphalt mixture within the ±7*L* range on the fiber outer sheath at 30 s (after stress dissipation). The increase in temperature during the creep phase leads to a rise in plastic deformation, particularly near the specimen’s top and bottom surfaces, influenced by boundary constraints. Within the central ±5*L* region of the grating, the plastic strain level of the asphalt mixture is nearly uniform but still exhibits a convex distribution—larger in the middle and smaller at the ends—which resembles the typical strain profile within a rut groove. However, the strain distribution on the outer surface within the effective length range is predominantly concave, forming a flattened “w”-shaped profile with a slight convexity at grating region. As the plastic strain level increases, the difference between the strain level in the 10 mm monitoring medium corresponding to the grating section and the strain levels at the adjacent positions at both ends continues to increase. This is reflected in Figure 12a, where the curve exhibits a more pronounced concave distribution near the 0 coordinate on the *x*-axis as the plastic strain increases. Under these conditions, compared to a uniform strain distribution, the strain from the adjacent positions at both ends contributes more to the strain in the grating section. This leads to a continuous increase in the strain transfer coefficient, potentially exceeding the strain level in the corresponding 10 mm monitoring medium. This distribution corresponds to a relatively high plastic strain transfer coefficient $\alpha_{p}$. Figure 12b shows the relationship among the plastic strain transfer coefficient $\alpha_{p}$, the viscoelastic strain transfer coefficient $\alpha_{ve}$, and the plastic strain $\varepsilon_{p}$. $\alpha_{p}$ is calculated at the moment following stress dissipation to ensure the transferred strain originates solely from plastic deformation, while $\alpha_{ve}$ is calculated from the strain increment in the 20th loading cycle. $\alpha_{p}$ reaches 95.19% at a low plastic strain level of 500 *με*, indicating the contribution of the high strain at the specimen ends to the grating region strain is still limited. However, as plastic strain increases, the high strain at both ends rapidly rises, gradually exceeding the central region, and when $\varepsilon_{p}$ exceeds 2300 *με*, the FBG monitored plastic strain becomes higher than that of the host medium. This evolution of $\alpha_{p}$ is determined by the flattened “w” shape of the plastic strain distribution. For rutting monitoring, the plastic strain at the rut groove sides is significantly higher than at the compressed rut bottom due to aggregate shear slip, forming a concave profile on the fiber surface and leading to higher strain transfer efficiency. Notably, the viscoelastic strain transfer coefficient decreases solely with increasing temperature and unaffected by the evolution of plastic strain. On the one hand, the effect of strain increments is independent of the existing strain state due to the elastic encapsulation layers; on the other hand, the viscoelastic and plastic behaviors of the asphalt mixture are decoupled, causing the plastic strain evolution to not influence the viscoelastic strain distribution along the outer sheath.

### 4.3. Strain Transfer Analysis of Fiber Bragg Grating in Pavement Base Layer

**Figure 13:**
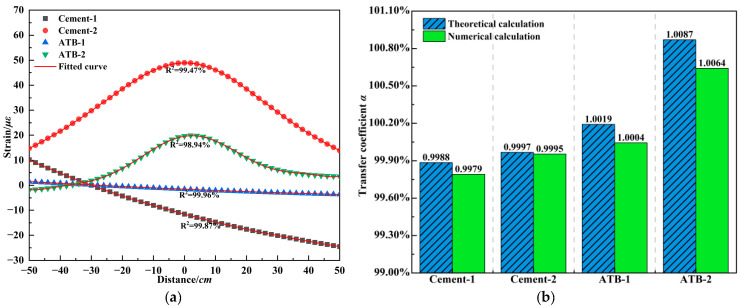
Strain analysis of embedded FBG in base layers under standard temperature. (**a**) Polynomial fitting of pavement strain distribution. (**b**) Strain transfer coefficient of embedded FBG.

Above research clarifies the strain transfer characteristics of armored FBG in asphalt mixtures. In pavement applications, FBG can be embedded at the bottom of the cement-treated base to monitor the fatigue performance or at the bottom of the asphalt-stabilized base to estimate the fatigue life of flexible base structures. The strain distribution on the outer surface significantly affects the strain level at the grating region. Given the varying stress mechanisms of pavement structures and materials, the representative pavement structures listed in Table 3 are adopted to analyze the FBG strain transfer. Meanwhile, a polynomial fitting approach is used to capture the strain distribution characteristics over a ±50 *L* around the grating region, and the theoretical calculation is compared with numerical simulation results for validation. Figure 13a presents the scatter plots and polynomial fits of strain distribution along the driving direction at the bottom of the asphalt-stabilized and cement-treated base layer under standard temperature. To ensure that the polynomial fitting accuracy is no less than 98%, a fifth-order polynomial function is used to fit the strain distribution on the outer surface of the FBG for the cement-treated base, while a ninth-order polynomial function is used for the asphalt-stabilized base. Polynomial fitting effectively captures the strain distribution within the road structure under vehicle movement. By utilizing the relationship given in Equation (12), the polynomial coefficients for the strain distribution in the fiber core segment are obtained, which are then used in Equation (7b) to derive the theoretical solution for the strain transfer coefficient in the grating region. Label 1 corresponds to the strain distribution when the vehicle is located 1 m away from the grating region, while Label 2 represents the condition when the vehicle is directly above the grating. Results show that the strain amplitude in the cement-treated base is higher than that in the asphalt-stabilized base, due to its deeper placement and ability to withstand more loading. Notably, when a vehicle is directly above the grating region, the strain distribution at the bottom of the asphalt-stabilized base is asymmetric, and a nearly constant strain zone is observed in the front region, which is attributed to viscoelastic effects that accumulate strain in the structure during vehicle movement. This is the reason why higher-order polynomial fitting is required for the ATB base layer. Such non-uniform strain distribution leads to variation in the strain transfer coefficient of the grating region depending on the vehicle load position. Figure 13b compares the strain transfer coefficient derived from the polynomial-based theoretical calculation and the numerical simulation under typical strain distribution for the two base types. The theoretical values are slightly lower than the numerical results, with the largest deviation of 0.23% observed in the asphalt-stabilized base. The transfer efficiency increases as the vehicle approaches the grating region. Moreover, the cement-treated base exhibits a strain transfer efficiency below 1, while the asphalt-stabilized base shows values greater than 1. This difference is attributed to the distinct material properties that the cement treated base behaves elastically without strain accumulation, while the viscoelasticity allows for strain accumulation, which contributes to a slightly higher strain level at the grating region compared to the host medium.

**Figure 14:**
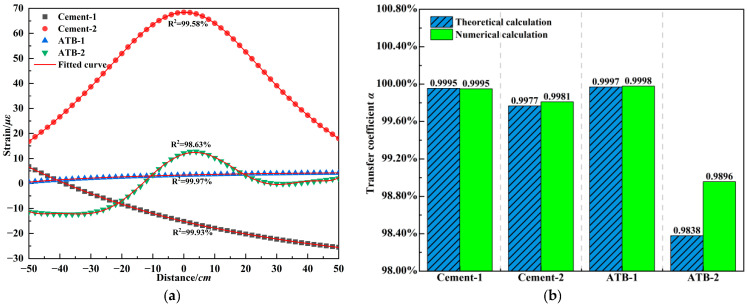
Strain analysis of embedded FBG in base layers under high temperature. (**a**) Polynomial fitting of pavement strain distribution. (**b**) Strain transfer coefficient of embedded FBG.

Since temperature is a key factor influencing the strain transfer coefficient, and considering the high temperature in summer, a linearly varying temperature field along the pavement depth is established. The asphalt surface temperature is set to 45 °C, with temperature gradients of 1 °C/cm in the upper and middle layers; 0.625 °C/cm is selected in the lower layer, while a constant temperature of 25 °C is applied to the asphalt-stabilized base (ATB). Figure 14a illustrates the strain distribution in the base layer under high-temperature conditions. Compared to the standard temperature field, the strain level at the bottom of the cement-treated base increased significantly. As the axle load approached the grating region, the surface strain on the sheath increased in compression, and a rise in tensile strain occurred when the load was directly above the grating region. The elevated temperature in the asphalt structures caused cement-treated base to withstand more stress, resulting in greater strain. In addition, the tensile strain in the asphalt-stabilized base significantly decreased, and the strain distribution along the sheath became more non-uniform. The intensified viscous behavior of asphalt mixtures at high temperatures led to an increased rate of strain accumulation in pavement regions not directly loaded by traffic, reflected in the upward-bending form of the ATB-2 strain profile. This also leads to a decrease in the accuracy of the polynomial fitting. However, the main fitting deviation occurs far from the grating region, and its impact on the strain transfer efficiency is further minimized. Notably, under high-temperature condition, the rate of strain attenuation at both ends of the grating region increased significantly, reducing the incremental strain contribution from the main strain-contributing section, thereby reducing the strain transfer coefficient. Figure 14b compares theoretical and numerical results of the strain transfer coefficient. The temperature rise caused a marked decline in the strain transfer efficiency of the asphalt-stabilized base and increased the variability in transfer efficiency across different axle positions. However, the effect on the cement-treated base was minimal. The polynomial-based theoretical model accurately predicted the strain transfer coefficient under various strain distribution patterns and multilayer packaging structures. In pavement structures, strain correction due to temperature variation is not critical for cement-treated base, while for asphalt-stabilized base, the temperature influence on strain transfer coefficient should be considered.

## 5. Conclusions

This study investigates the factors influencing the strain transfer efficiency of embedded FBG in asphalt pavements. FBG strain distribution is fitted using a polynomial function, addressing the boundary condition requirements of embedded FBG. A numerical simulation method is employed to investigate the evolution of strain transfer efficiency under dynamic load response and permanent deformation in asphalt mixtures, which can provide data correction for long-term strain monitoring of asphalt pavements. The sensitivity analysis of the encapsulation layers obtained in this study offers guidance for the structural enhancement design of multilayer encapsulated FBG. The main conclusions are as follows:

(1) The bond length is a key factor affecting the strain transfer efficiency of FBG. For embedded FBG, the actual bond length is the full length of the fiber; however, the contribution of strain from sections far from the grating region to the transfer coefficient significantly decreases. Therefore, a contribution rate threshold of 5% is used as the criterion to determine the effective bond length. For the braided metal armor FBG used in this study, the effective bonding length is 14 cm.
(2) The outer sheath is the main internal factor affecting strain transfer efficiency. An increase in modulus or a decrease in thickness significantly enhances the strain transfer coefficient. Considering both the survival of embedded FBG and the sensitivity of monitoring, it is recommended to use high-modulus materials for the encapsulation layer while controlling the structural thickness to within 0.5 mm, thereby ensuring that the strain transfer coefficient does not below 95%.
(3) FBG can capture the viscoelastic response behavior effectively within asphalt mixtures, and temperature is the major external factor affecting strain transfer efficiency. In particular, under high-temperature conditions in summer, the strain transfer coefficient of the FBG decreases by approximately 1.21% compared to a standard temperature of 20 °C. Under typical vehicle speeds, changes in load frequency have a negligible effect on the strain transfer coefficient.
(4) When performing long-term strain monitoring on the pavement surface layer, the effect of plastic strain magnitude and distribution on the FBG strain transfer coefficient must be considered. The permanent deformation loading simulation results for cylindrical specimens show the increase in plastic strain enhances the FBG strain transfer coefficient, owing to the concave strain distribution. When the grating region is placed in the rut, the strain transfer coefficient can be improved by adjusting based on the monitored strain level.
(5) The proposed polynomial fitting method eliminates the assumption boundary conditions during the differential equation solution, making it more consistent with the actual situation of embedded FBG. A fifth-order polynomial function is sufficient for fitting the strain distribution in cement-treated base, while a ninth-order polynomial function is required for asphalt-stabilized base due to the presence of viscoelastic effects. The results from the pavement numerical analysis model validate the accuracy of the strain transfer coefficient obtained through this polynomial fitting method.

## Figures and Tables

**Table 1:** Basic parameters of the braided metal armor FBG.

Structural Layer	Outer Diameter/mm	Young’s Modulus/Gpa	Poisson’s Ratio
Optical fiber	0.125 (2*r*_0_)	72	0.2
Coating layer	0.4 (2*r*_1_)	10	0.25
Adhesive layer	1.2 (2*r*_2_)	2	0.3
Braided steel mesh	1.7 (2*r*_3_)	195	0.3
PVC outer sheath	2.7 (2*r*_4_)	0.28	0.4

**Table 2:** Thermoviscoelastic parameters of AC-20.

$E_{\infty }$/Mpa	$E_{1}$	$E_{2}$	$E_{3}$	$E_{4}$	$E_{5}$	$E_{6}$	$E_{7}$	$E_{8}$
853.07	3214.52	5638.87	7184.66	8071.30	7549.23	3881.01	1906.39	2.82
$\rho_{\infty }$/*S*	$\rho_{1}$	$\rho_{2}$	$\rho_{3}$	$\rho_{4}$	$\rho_{5}$	$\rho_{6}$	$\rho_{7}$	$\rho_{8}$
∞	10^−5^	10^−4^	10^−3^	10^−2^	10^−1^	10^0^	10^1^	10^2^
$\theta_{ve}$	19,136.4	$T_{ref}$/°C	25					

**Table 3:** Structural parameters of the typical asphalt-stabilized base pavement.

Structural Layer	Thickness/cm	Modulus/Mpa	Poisson’s Ratio
AC13	4	Prony1	0.25
AC16	6	Prony2	0.25
AC20	8	Prony3	0.25
ATB25	12	Prony4	0.25
Cement-treated	20	10,000	0.35
Subgrade	200	80	0.4

**Table 4:** Mohr–Coulomb plasticity model parameters for asphalt mixtures.

Yield Parameters	Flow Parameters		Hardening Parameters			Temperature Parameters		
ϕ/rad	$\Gamma^{vp}$/10^−16^	N	$c_{0}$/kpa	$c_{1}$/kap	$c_{2}$/kpa	$T_{ref}$/°C	$\theta_{vp}$	$\theta_{c}$
0.2	3.18	2.4	82	354.7	−250	50	−13,790	4152

## Data Availability

Dataset available on request from the authors. The raw data supporting the conclusions of this article will be made available by the authors on request.
